# Integration of Telemedicine Consultation Into a Tertiary Radiation Oncology Department: Predictors of Use, Treatment Yield, and Effects on Patient Population

**DOI:** 10.1200/CCI.23.00239

**Published:** 2024-04-17

**Authors:** Yasamin Sharifzadeh, William G. Breen, William S. Harmsen, Adam C. Amundson, Allison E. Garda, David M. Routman, Mark R. Waddle, Kenneth W. Merrell, Christopher L. Hallemeier, Nadia N. Laack, Anantha Kollengode, Kimberly S. Corbin

**Affiliations:** ^1^ Department of Radiation Oncology, Mayo Clinic, Rochester, MN; ^2^ Division of Biomedical Statistics and Informatics, Mayo Clinic, Rochester, MN

## Abstract

**PURPOSE:**

The COVID-19 pandemic led to rapid expansion of telemedicine. The implications of telemedicine have not been rigorously studied in radiation oncology, a procedural specialty. This study aimed to evaluate the characteristics of in-person patients (IPPs) and virtual patients (VPs) who presented to a large cancer center before and during the pandemic and to understand variables affecting likelihood of receiving radiotherapy (yield) at our institution.

**METHODS:**

A total of 17,915 patients presenting for new consultation between 2019 and 2021 were included, stratified by prepandemic and pandemic periods starting March 24, 2020. Telemedicine visits included video and telephone calls. Area deprivation indices (ADIs) were also compared.

**RESULTS:**

The overall population was 56% male and 93% White with mean age of 63 years. During the pandemic, VPs accounted for 21% of visits, were on average younger than their in-person (IP) counterparts (63.3 years IP *v* 62.4 VP), and lived further away from clinic (215 miles IP *v* 402 VP). Among treated VPs, living closer to clinic was associated with higher yield (odds ratio [OR], 0.95; *P* < .001). This was also seen among IPPs who received treatment (OR, 0.96; *P* < .001); however, the average distance from clinic was significantly lower for IPPs than VPs (205 miles IP *v* 349 VP). Specialized radiotherapy (proton and brachytherapy) was used more in VPs. IPPs had higher ADI than VPs. Among VPs, those treated had higher ADI (*P* < .001).

**CONCLUSION:**

Patient characteristics and yield were significantly different between IPPs and VPs. Telemedicine increased reach to patients further away from clinic, including from rural or health care–deprived areas, allowing access to specialized radiation oncology care. Telemedicine has the potential to increase the reach of other technical and procedural specialties.

## Teaser

Telemedicine allows patients who live further away and/or in health care–deprived areas to access radiation therapy.

## INTRODUCTION

Telehealth, telemedicine, and related terms are defined by the Centers for Medicare and Medicaid Services as services that a physician or practitioner provides via two-way, interactive technology that substitutes for an in-person (IP) visit.^1^ The use of telemedicine rapidly expanded during the COVID-19 pandemic as many health care facilities were forced to halt IP appointments. Telemedicine became integral to ensuring timely, continued care for patients with nonelective medical needs such as cancer.

**CONTEXT**

**Key Objective**
What were the characteristics of in-person patients (IPPs) and virtual patients (VPs) who presented to a large cancer center before and during the pandemic and how did telemedicine affect the likelihood of undergoing radiotherapy (yield)?
**Knowledge Generated**
In both VPs and IPPs, proximity to clinic was associated with higher yield and higher area deprivation index while VPs received more proton radiation and brachytherapy.
**Relevance *(J.L. Warner)***
This study describes the impact of telemedicine in the context of radiation oncology, telemedicine allowed for increased reach to rural patients.*
*Relevance section written by *JCO Clinical Cancer Informatics* Editor-in-Chief Jeremy L. Warner, MD, MS, FAMIA, FASCO.

Before the pandemic, only 15%-20% of physicians reported experience with telemedicine.^2^ As the pandemic accelerated the need for remote medical care, there was a 154% increase in the use of telemedicine in the United States in the first quarter of 2020 when compared with the first quarter of 2019.^3^ This was driven by the need for health care during social isolation and supported by policy changes, including the declaration of a public health emergency (PHE) in January 2020. The PHE waived previous telemedicine regulations and allowed for easier implementation and access.^4^ Since then, there has been a sustained growth in the use of telemedicine.^5^

The impact of telemedicine on access to oncology care has yet to be fully elucidated, especially in the highly specialized, technical, and procedural field of radiation oncology. Hsiao et al found radiation oncology to be the fourth lowest utilizer of telemedicine among 45 specialties between 2020 and 2021.^6^ Comparing a population of radiation oncology patients presenting IP before the COVID-19 pandemic with those presenting virtually during the pandemic, one study found telemedicine users to be younger, female, and with higher performance status.^7^ Another study evaluating disparities in telemedicine use in a large cancer care network found race, ethnicity, income, and type of insurance most influential to telemedicine utilization.^8^ Given the limited understanding of telemedicine in the radiation oncology setting, we aimed to not only further characterize patient utilization of telemedicine in a large radiation oncology center in the United States, but also to understand if the use of telemedicine affected access to radiation oncology care and the likelihood of receiving radiation at our center.

## METHODS

### Selection and Description of Patients

We conducted a retrospective, observational study within a large tertiary radiation oncology center in the midwestern United States. All new patient consultations between January 2019 and December 2021 were identified. Before March 2020, all consultations were performed IP. Telemedicine consultations, which started in March 24, 2020, included video conferencing technology or telephone calls. The term telemedicine is used interchangeably with virtual patients (VPs) in this study. A total of 17,915 patients were included. This study was approved by the institutional review board.

Demographic, medical, and treatment variables were extracted from the electronic medical record. Medicare Advantage plans were coded as private insurance, while primary Medicare/Medicaid plans were coded as public insurance. If a patient had multiple consultations in the study time range, only their first consultation was included. Miles from clinic, also referred to as distance traveled to clinic or patient distance from clinic, was the distance between a patient's primary address and the radiation oncology center. The clinic and treatment center were at the same location. Those with a primary US address were labeled as US residents. Radiation treatment modalities included photon, proton, brachytherapy, Gamma Knife, and other. The other treatment modalities included intraoperative radiation therapy, orthovoltage, and electron radiotherapy. Treatment yield was defined by whether a patient received radiation treatment at our center by December 31, 2021.

The area deprivation index (ADI), as developed by the Health Resources and Services Administration and Kind et al,^9^ was used to assess the national health care deprivation percentiles of patients on the basis of zip code.^10,11^ A higher ADI number indicates higher health care deprivation. To better understand the population of patients served by our clinic, we used a map of county populations by state provided by the 2020 US Census.^12^

### Statistical Analysis

A mean (standard deviation) and number (proportion) are reported for continuous and categorical variables, respectively.

Statistical analyses included comparing demographic and treatment variables by type of visit (IP *v* virtual), yield, and prepandemic versus during the pandemic (starting March 24, 2020). Sex, race, ethnicity, US residency, insurance type (private *v* public), visit type, consultation year, primary diagnosis, categorized and mean miles from clinic, and treatment modality were assessed for association with yield. A chi square test was used to compare discrete unordered variables, a Wilcoxon rank-sum test (comparison between two groups) or Kruskal-Wallis test (comparison among more than two groups) was used to compare ordered categorical variables, and a *T*-test assuming equal variances was used for continuous variables (age and mean miles from clinic). Multivariable logistic modeling was used to investigate associations between variables of interest and likelihood for virtual visit and yield. Variables of interest included in the logistic modeling were chosen if they were thought to provide clinical significance in the context of our data. For example, although univariate analysis may have shown race to be significantly different between two groups, our racial distribution was so homogeneous that we did not believe inclusion into our modeling would yield clinically significant results. We also believed many of our variables to be correlated with each other (sex and primary diagnosis, US residency status and miles from clinic, race and ethnicity within our population) and therefore only one of these variables were included.

The alpha level was set at *P* < .05 for statistical significance. SAS version 9.4 was used for analysis.

## RESULTS

### Overall Patient Characteristics (January 2019-December 2021)

Table 1 displays patient characteristics for the entire population before and during the pandemic. In the study period, 15,977 (88%) patients were seen IP. Treatment yield for all patients was 69%. Most patients were male (56%). Mean age was 63 years (IQR, 55.8-73.1). Most had private insurance (58%). Ninety-three percent were White and 98% were non-Hispanic. Most common malignancies were genitourinary (GU; 25%), breast (14%), and GI (11%). Although 44% of patients lived within 100 miles of clinic, the mean distance traveled to clinic was 260 miles (IQR, 64-291 miles).

**TABLE 1.** Demographics and Medical Characteristics Stratified by Prepandemic and Pandemic Periods (N = 17,915)

Demographic/Medical Characteristic	Prepandemic (n = 7,561)	Pandemic (n = 10,354)	Total (N = 17,915)	*P*
Sex, No. (%)				
Female	3,309 (43.8)	4,503 (43.5)	7,812 (43.6)	.65
Male	4,252 (56.2)	5,850 (56.5)	10,102 (56.4)	
Nonbinary	0 (0.0)	1 (0.0)	1 (0.0)	
Race, No. (%)				
White	6,924 (93.1)	9,537 (93.4)	16,461 (93.1)	.15
African American/Black	126 (1.7)	210 (2.1)	336 (1.9)	
Asian	160 (2.2)	205 (2.0)	365 (2.1)	
Native American	33 (0.4)	44 (0.4)	77 (0.4)	
Pacific Islander	4 (0.1)	7 (0.1)	11 (0.1)	
Multiracial	187 (2.5)	207 (2.0)	394 (2.2)	
Missing	127	144	271	
Ethnicity, No. (%)				
Hispanic/Latino	174 (2.4)	212 (2.1)	386 (2.2)	.24
Non-Hispanic/Latino	7,174 (97.6)	9,858 (97.9)	17,032 (97.8)	
Missing	213	284	497	
Age, years				
Mean (SD)	62.8 (15.1)	63.1 (15.2)	63.0 (15.2)	.19
Residence, No. (%)				
US	7,364 (97.4)	10,182 (98.3)	17,546 (97.9)	<.001
Non-US	197 (2.6)	72 (0.7)	269 (1.5)	
Insurance, No. (%)				
Private	4,222 (55.8)	6,116 (59.1)	10,338 (57.7)	<.001
Public	3,339 (44.2)	4,238 (40.9)	7,577 (42.3)	
Primary diagnosis, No. (%)				
CNS	738 (9.8)	1,035 (10.0)	1,773 (9.9)	.07
Head, neck, skin	706 (9.3)	940 (9.1)	1,646 (9.2)	
Thoracic	588 (7.8)	754 (7.3)	1,342 (7.5)	
Breast	1,039 (13.7)	1,424 (13.8)	2,463 (13.7)	
GI	867 (11.5)	1,182 (11.4)	2,049 (11.4)	
Sarcoma/bone	268 (3.5)	347 (3.4)	615 (3.4)	
GU	1,812 (24.0)	2,680 (25.9)	4,492 (25.1)	
Gyn	291 (3.8)	407 (3.9)	698 (3.9)	
Lymphoma	222 (2.9)	310 (3.0)	532 (3.0)	
Pediatric	161 (2.1)	236 (2.3)	397 (2.2)	
Palliative	765 (10.1)	907 (8.8)	1,672 (9.3)	
Benign	104 (1.4)	132 (1.3)	236 (1.3)	
Consultation year, No. (%)				
2019	6,120 (80.9)	0 (0.0)	6,120 (34.2)	—
2020	1,441 (19.1)	4,352 (42.0)	5,793 (32.3)	
2021	0 (0.0)	6,002 (58.0)	6,002 (33.5)	
Miles from clinic, No. (%)				
0-99	3,200 (43.5)	4,520 (44.4)	7,720 (44.0)	.13
100-199	1,234 (16.8)	1,793 (17.6)	3,027 (17.3)	
200-299	1,080 (14.7)	1,433 (14.1)	2,513 (14.3)	
300-399	485 (6.6)	665 (6.5)	1,150 (6.6)	
400-499	288 (3.9)	408 (4.0)	696 (4.0)	
≥500	1,073 (14.6)	1,358 (13.3)	2,431 (13.9)	
Missing	201	177	378	
Miles from clinic				
Mean (SD)	267.9 (372.3)	254.2 (360.3)	259.9 (365.4)	.01
Visit type, No. (%)				
In-person	7,561 (100)	8,211 (79.3)	15,772 (88.0)	—
Virtual	0	2,143 (20.7)	2,143 (12.0)	
Treatment, No. (%)				
Yes	5,050 (66.8)	6,909 (66.7)	11,959 (66.8)	.93
No	2,511 (33.2)	3,445 (33.3)	5,956 (33.2)	

Abbreviations: GU, genitourinary; SD, standard deviation.

During the pandemic, VP accounted for 21% of visits, non-US resident patient consultations decreased, the use of private insurance increased, and patients' average miles from clinic decreased. Treatment yield was not affected by the pandemic.

### Patient Characteristics During the Pandemic (March 2020-December 2021)

Table 2 shows differences between IPPs and VPs during the pandemic only. On univariate analysis, VPs were more likely to be male, younger in age, present from further distances, and were 1.5 times less likely to receive treatment at our institution (yield: 48% VPs *v* 72% IPPs). A significantly higher portion of VPs presented for GU diagnoses (43% VPs *v* 21% IPPs) and a significantly lower portion presented for palliative diagnosis (2.8% VPs *v* 10% IPPs). On multivariable logistic modeling, only age and distance from clinic remained significantly different between IPPs and VPs during the pandemic; VPs were more likely to be younger in age and live farther from clinic.

**TABLE 2.** Demographic and Medical Characteristics by Visit Type During the Pandemic (n = 10,354)

Demographic/Medical Characteristic	In-Person (n = 8,211)	Virtual (n = 2,143)	Total (n = 10,354)	Univariate *P*	Multivariable *P*	Odds Ratio (95% CI)
Sex, No. (%)						
Female	3,770 (45.9)	733 (34.2)	4,503 (43.5)	<.001	.53	1.05 (0.91 to 1.21)
Male	4,440 (54.1)	1,410 (65.8)	5,850 (56.5)			
Nonbinary	1 (0.0)	0 (0.0)	1 (0.0)			
Race, No. (%)						
White	7,577 (93.3)	1,960 (93.7)	9,537 (93.4)	.26		
African American/Black	167 (2.1)	43 (2.1)	210 (2.1)			
Asian	157 (1.9)	48 (2.3)	205 (2.0)			
Native American	40 (0.5)	4 (0.2)	44 (0.4)			
Pacific Islander	5 (0.1)	2 (0.1)	7 (0.1)			
Multiracial	172 (2.1)	35 (1.7)	207 (2.0)			
Missing	93	51	144			
Ethnicity, No. (%)						
Hispanic/Latino	163 (2.0)	49 (2.4)	212 (2.1)	.33		
Non-Hispanic/Latino	7,848 (98.0)	2,010 (97.6)	9,858 (97.9)			
Missing	200	84	284			
Age, years						
Mean (SD)	63.3 (15.2)	62.4 (15.2)	63.1 (15.2)	.02	<.001	0.86 (0.82 to 0.91)
Residence, No. (%)						
US	8,074 (98.3)	2,108 (98.4)	10,182 (98.3)	.91		
Non-US	137 (1.7)	35 (1.6)	172 (1.7)			
Insurance, No. (%)						
Private	4,833 (58.9)	1,283 (59.9)	6,116 (59.1)	.40		
Public	3,378 (41.1)	860 (40.1)	4,238 (40.9)			
Primary diagnosis, No. (%)						
CNS	831 (10.1)	204 (9.5)	1,035 (10.0)	<.001		
Head, neck, skin	784 (9.5)	156 (7.3)	940 (9.1)			
Thoracic	631 (7.7)	123 (5.7)	754 (7.3)			
Breast	1,158 (14.1)	266 (12.4)	1,424 (13.8)			
GI	977 (11.9)	205 (9.6)	1,182 (11.4)			
Sarcoma/bone	283 (3.4)	64 (3.0)	347 (3.4)			
GU	1,760 (21.4)	920 (42.9)	2,680 (25.9)			
Gyn	370 (4.5)	37 (1.7)	407 (3.9)			
Lymphoma	269 (3.3)	41 (1.9)	310 (3.0)			
Pediatric	184 (2.2)	52 (2.4)	236 (2.3)			
Palliative	846 (10.3)	61 (2.8)	907 (8.8)			
Benign	118 (1.4)	14 (0.7)	132 (1.3)			
Consultation year, No. (%)						
2020	3,542 (43.1)	810 (37.8)	4,352 (42.0)	<.001		
2021	4,669 (56.9)	1,333 (62.2)	6,002 (58.0)			
Miles from clinic, No. (%)						
0-99	4,037 (50.0)	483 (22.9)	4,520 (44.4)	<.001		
100-199	1,442 (17.9)	351 (16.7)	1,793 (17.6)			
200-299	1,023 (12.7)	410 (19.4)	1,433 (14.1)			
300-399	456 (5.7)	209 (9.9)	665 (6.5)			
400-499	268 (3.3)	140 (6.6)	408 (4.0)			
≥500	843 (10.4)	515 (24.0)	1,358 (13.3)			
Missing	142	35	177			
Miles from clinic						
Mean (SD)	215.4 (326.0)	402.4 (438.4)	254.2 (360.3)	<.001	<.001	1.12 (1.11 to 1.14)
Treatment, No. (%)						
Yes	5,883 (71.6)	1,026 (47.9)	6,909 (66.7)	<.001		
No	2,328 (28.4)	1,117 (52.1)	3,445 (33.3)			
Treatment modality,^a^ No. (%)						
Photon	3,542 (43.1)	529 (24.7)	4,071 (58.9)	<.001		
Proton	1,361 (16.6)	400 (18.7)	1,761 (17.0)			
Brachytherapy	242 (2.9)	58 (2.7)	300 (2.9)			
Gamma Knife	605 (7.4)	21 (1.0)	626 (6.0)			
Other	133 (1.6)	18 (0.8)	151 (1.5)			
Unknown/missing	2,328 (28.4)	1,117 (52.1)	3,445 (33.3)			

Abbreviations: GU, genitourinary; SD, standard deviation.

a Treatment modality of those who did not receive treatment at our institution was not known and therefore removed from statistical analysis but included in the percentages shown.

### Patient Characteristics by Yield During the Pandemic (March 2020-December 2021)

#### IPPs

A total of 8,211 patients were seen IP during the pandemic, of whom 5,883 (72%) underwent treatment at our institution (Table 3). On univariate analysis, those who received treatment were younger and lived closer to the clinic. We did not appreciate sex or insurance differences on univariate analysis. Patients who self-reported as White or multiracial were the most likely to undergo treatment. By disease category, those with benign, pediatric, and CNS diagnoses were most likely to undergo treatment. Multivariable logistic modeling found male sex and fewer miles from clinic to be associated with higher yield in IPPs, but age was no longer a significant factor.

**TABLE 3.** In-Person Patient Characteristics During the Pandemic by Yield (n = 8,211)

Demographic/Medical Characteristic	Treatment	
	Yes (n = 5,883)	No (n = 2,328)	Total (n = 8,211)	Univariate *P*	Multivariable *P*	Odds Ratio (95% CI)
Sex, No. (%)						
Female	2,735 (72.5)	1,035 (27.5)	3,770 (45.9)	.20	.02	0.85 (0.75 to 0.98)
Male	3,147 (70.9)	1,293 (29.1)	4,440 (54.1)			
Nonbinary	1 (100.0)	0 (0.0)	1 (0.0)			
Race, No. (%)						
White	5,472 (72.2)	2,105 (27.8)	7,577 (93.3)	.004		
African American/Black	109 (65.3)	58 (34.7)	167 (2.1)			
Asian	99 (63.1)	58 (36.9)	157 (1.9)			
Native American	30 (75.0)	10 (25.0)	40 (0.5)			
Pacific Islander	1 (20.0)	4 (80.0)	5 (0.1)			
Multiracial	119 (69.2)	53 (30.8)	172 (2.1)			
Missing	53 (57.0)	40 (43.0)	93			
Ethnicity, No. (%)						
Hispanic/Latino	119 (73.0)	44 (27.0)	163 (2.0)	.75		
Non-Hispanic/Latino	5,642 (71.9)	2,206 (28.1)	7,848 (98.0)			
Missing	122 (61.0)	78 (39.0)	200			
Age, years						
Mean (SD)	62.7 (15.6)	64.6 (14.0)	63.3 (15.3)	<.001	.84	1.00 (0.96 to 1.04)
Residence, No. (%)						
US	5,785 (71.6)	2,289 (28.4)	8,074 (98.3)	.98		
Non-US	98 (71.5)	39 (28.5)	137 (1.7)			
Insurance, No. (%)						
Private	3,478 (72.0)	1,355 (28.0)	4,833 (58.9)	.45		
Public	2,405 (71.2)	973 (28.8)	3,378 (41.1)			
Primary diagnosis, No. (%)						
CNS	705 (84.8)	126 (15.2)	831 (10.1)	<.001		
Head, neck, skin	474 (60.5)	310 (39.5)	784 (9.5)			
Thoracic	434 (68.8)	197 (31.2)	631 (7.7)			
Breast	744 (64.2)	414 (35.8)	1,158 (14.1)			
GI	724 (74.1)	253 (25.9)	977 (11.9)			
Sarcoma/bone	217 (76.7)	66 (23.3)	283 (3.4)			
GU	1,113 (63.2)	647 (36.8)	1,760 (21.4)			
Gyn	300 (81.1)	70 (18.9)	370 (4.5)			
Lymphoma	226 (84.0)	43 (16.0)	269 (3.3)			
Pediatric	158 (85.9)	26 (14.1)	184 (2.2)			
Palliative	681 (80.5)	165 (19.5)	846 (10.3)			
Benign	107 (90.7)	11 (9.3)	118 (1.4)			
Miles from clinic, No. (%)						
0-99	3,083 (76.4)	954 (23.6)	4,037 (50.0)	<.001		
100-199	1,007 (69.8)	435 (30.2)	1,442 (17.9)			
200-299	655 (64.0)	368 (36.0)	1,023 (12.7)			
300-399	289 (63.4)	167 (36.6)	456 (5.7)			
400-499	188 (70.1)	80 (29.9)	268 (3.3)			
≥500	560 (66.4)	283 (33.6)	843 (10.4)			
Missing	101 (71.1)	41 (28.9)	142			
Miles from clinic						
Mean (SD)	204.5 (329.3)	243.1 (315.9)	215.4 (326.0)	<.001	<.001	0.96 (0.95 to 0.97)
Treatment modality,^a^ No. (%)						
Photon	3,542 (60.2)	NA	3,542 (43.1)	—		
Proton	1,361 (23.1)	NA	1,361 (16.6)			
Brachytherapy	242 (4.1)	NA	242 (2.9)			
Gamma Knife	605 (10.3)	NA	605 (7.4)			
Other	133 (2.3)	NA	133 (1.6)			
Unknown	0 (0.0)	2,328 (100)	2,328 (28.4)			

Abbreviations: GU, genitourinary; NA, not available; SD, standard deviation.

a Treatment modality of those who did not receive treatment at our institution was not known and therefore removed from statistical analysis but included in the percentages shown.

#### VPs

There were 2,143 new VP consultations, of whom 1,026 (48%) underwent treatment at our institution (Table 4). In contrast to the IP population, likelihood of treatment was not affected by sex. On univariate analysis, younger patients and those presenting from closer distances were more likely to undergo treatment; however, these distances were still significantly further away than their IP counterparts (205 miles IP *v* 349 miles VP). Lymphoma, pediatric, and benign diagnoses had the highest yields, albeit significantly lower than their IP counterparts. Upon multivariable logistic modeling, miles from clinic was the only factor contributing to the likelihood of VPs undergoing treatment at our institution, with patients living closer to clinic being more likely to receive treatment at the facility.

**TABLE 4.** Virtual Patient Characteristics During the Pandemic by Yield (n = 2,143)

Demographic/Medical Characteristic	Treatment	
	Yes (n = 1,026)	No (n = 1,117)	Total (n = 2,143)	Univariate *P*	Multivariable *P*	Odds Ratio (95% CI)
Sex, No. (%)						
Female	350 (47.7)	383 (52.3)	733 (34.2)	.93	.35	0.88 (0.68 to 1.15)
Male	676 (47.9)	734 (52.1)	1,410 (65.8)			
Nonbinary	0 (0.0)	0 (0.0)	0 (0.0)			
Race, No. (%)						
White	955 (48.7)	1,005 (51.3)	1,960 (93.7)	.52		
African American/Black	21 (48.8)	22 (51.2)	43 (2.1)			
Asian	19 (39.6)	29 (60.4)	48 (2.3)			
Native American	1 (25.0)	3 (75.0)	4 (0.2)			
Pacific Islander	1 (50.0)	1 (50.0)	2 (0.1)			
Multiracial	13 (37.1)	22 (62.9)	35 (1.7)			
Missing	16 (31.4)	35 (68.6)	51			
Ethnicity, No. (%)						
Hispanic/Latino	23 (46.9)	26 (53.1)	49 (2.4)	.81		
Non-Hispanic/Latino	979 (48.7)	1,031 (51.3)	2,010 (97.6)			
Missing	24 (28.6)	60 (71.4)	84			
Age, years						
Mean (SD)	61.5 (16.2)	63.3 (14.2)	62.4 (15.2)	.006	.06	0.93 (0.87 to 1.00)
Residence, No. (%)						
US	1,021 (48.4)	1,087 (51.6)	2,108 (98.4)	<.001		
Non-US	5 (14.3)	30 (85.7)	35 (1.6)			
Insurance, No. (%)						
Private	617 (48.1)	666 (51.9)	1,283 (59.9)	.81		
Public	409 (47.6)	451 (52.4)	860 (40.1)			
Primary diagnosis, No. (%)						
CNS	99 (48.5)	105 (51.5)	204 (9.5)	.01		
Head, neck, skin	67 (42.9)	89 (57.1)	156 (7.3)			
Thoracic	61 (49.6)	62 (50.4)	123 (5.7)			
Breast	120 (45.1)	146 (54.9)	266 (12.4)			
GI	109 (53.2)	96 (46.8)	205 (9.6)			
Sarcoma/bone	24 (37.5)	40 (62.5)	64 (3.0)			
GU	423 (46.0)	497 (54.0)	920 (42.9)			
Gyn	22 (59.5)	15 (40.5)	37 (1.7)			
Lymphoma	27 (65.9)	14 (34.1)	41 (1.9)			
Pediatric	34 (65.4)	18 (34.6)	52 (2.4)			
Palliative	31 (50.8)	30 (49.2)	61 (2.8)			
Benign	9 (64.3)	5 (35.7)	14 (0.7)			
Miles from clinic, No. (%)						
0-99	294 (60.9)	189 (39.1)	483 (22.9)	<.001		
100-199	190 (54.1)	161 (45.9)	351 (16.7)			
200-299	195 (47.6)	215 (52.4)	410 (19.4)			
300-399	92 (44.0)	117 (56.0)	209 (9.9)			
400-499	56 (40.0)	84 (60.0)	140 (6.6)			
≥500	194 (37.7)	321 (62.3)	515 (24.4)			
Missing	5 (14.3)	30 (85.7)	35			
Miles from clinic						
Mean (SD)	349.4 (420.1)	452.2 (449.3)	402.4 (438.5)	<.001	<.001	0.95 (0.93 to 0.97)
Treatment modality,^a^ No. (%)						
Photon	529 (51.6)	NA	529 (24.7)	—		
Proton	400 (39.0)	NA	400 (18.7)			
Brachytherapy	58 (5.7)	NA	58 (2.7)			
Gamma Knife	21 (2.0)	NA	21 (1.0)			
Other	18 (1.8)	NA	18 (0.8)			
Unknown	0	1,117 (100)	1,117 (52.1)			

Abbreviations: GU, genitourinary; NA, not available; SD, standard deviation.

a Treatment modality of those who did not receive treatment at our institution was not known and therefore removed from statistical analysis but included in the percentages shown.

Compared with IPPs, VPs were 1.9 times more likely to receive proton radiation (23% IP *v* 44% VP) and had slightly higher use of brachytherapy (4% IP *v* 6% VP). Proton use for lymphoma and CNS VPs were 2.5 and 2.2 times higher than in IPPs, respectively. GU malignancies accounted for the largest absolute number of VP treatments (188 VPs treated).

### Census and ADI

Appendix Figure A1 shows a map from the US Census Bureau of counties by persons per square mile, with circles representing incremental 100-mile radii away from the clinic. Apart from one large metropolitan area at the edge of the 100-mile radius, most surrounding counties can be classified as rural, with <500 persons per square mile.^13^ As seen in Table 1, over 60% of all patients presented from within 200 miles of the clinic.

Appendix Figure A2 shows the national ADI for Minnesota and its surrounding states, with red representing the most disadvantaged group. Those presenting IP had significantly higher ADI than those presenting virtually (Table 5). In both IPPs and VPs, those who received treatment had significantly higher ADIs (Appendix Tables A1 and A2). In fact, those who presented IP and underwent treatment had the highest ADI. However, the difference in ADI by yield was most significant in VPs (46 *v* 43).

**TABLE 5.** ADI by Type of Visit During the Pandemic

ADI Characteristic	In-Person (n = 8,211)	Virtual (n = 2,143)	*P*
ADI, No. (%)			
Available	7,556 (92.0)	1,900 (88.7)	
US, unavailable	518 (6.3)	208 (9.7)	
International	137 (0.9)	35 (1.6)	
ADI, national percentage			
Mean (SD)	49.4 (22.4)	44.5 (24.0)	<.001

Abbreviations: ADI, area deprivation index; SD, standard deviation.

## DISCUSSION

The COVID-19 pandemic elevated telemedicine to the forefront of medical practice. Despite rapid adoption, the impact of telemedicine on access to cancer care, especially to procedural specialties such as radiation oncology, remains unclear. In our study of 17,915 new patient consultations seen from 2019 to 2021 in a tertiary radiation oncology clinic, we aimed to understand the impact of rapid telemedicine implementation on patient demographics and treatment yield and telemedicine's potential to increase access to radiotherapy. Although VPs were on average younger and lived farther than their IP counterparts, only distance from clinic affected the likelihood of treatment for VPs, with patients who elected to undergo treatment living on average closer than those who did not. Similarly, IPPs were more likely to undergo treatment if they were men and living closer to the clinic. Although treatment yield was higher in the VPs living closer, they remained statistically further from the clinic compared with IPPs. Compared with VPs who did not undergo treatment, VPs undergoing treatment had a higher ADI. These findings suggest that telemedicine was effective in increasing access to specialized radiotherapy for patients living further away, especially those with higher ADI and potentially less access to specialized treatment near their home.

At the onset of the pandemic, US ambulatory clinics reported up to 70% reduction in outpatient visits with variable patient volume recovery by 2021.^14-17^ One study found that almost 41% of patients with cancer experienced decreases in total visits and up to 52% reported decreased IP visits.^15^ Another study found that 20% of patients with cancer reported a delay in their cancer care.^18^ In comparison, our department experienced just a 5% reduction in new patient volume in 2020, possibly related to swift adoption of telemedicine by the institution and patients.^19^ As our institution adapted to the pandemic, new patient consultations in 2021 returned to prepandemic numbers and 22% of visits remained virtual. Our VPs were on average younger and were more likely to be male. In 2021, the CDC found that 37% of adults used telemedicine within the preceding year with higher use reported by females, those older than 65 years, and Non-Hispanic American Indian or Alaskan Natives.^20^ By contrast, a report by the Department of Health and Human Services found the highest telemedicine utilizers to be young adults and White patients, with the least utilization by people older than 65 years, and Black, Asian, and Latino populations.^21^ Like our study, a medical oncology outpatient cancer with 29% virtual visits in 2020 reported higher utilization among non-Hispanic White patients.^8^ The differences in the use of telemedicine in the general population and those presenting for radiotherapy may be at least partially attributable to the older demographic of patients with cancer (who may be less privy to using telemedicine),^22^ the increase in men presenting for GU diagnoses, as well as our large catchment of nonmetropolitan patients. Our data showed that VPs presented more frequently for GU diagnoses and less often for palliative consultations. Similarly, a small study reviewing patient satisfaction with telemedicine in radiotherapy reported more use of telemedicine in younger patients and among those presenting for GU and less for palliative diagnoses.^7^ Although this may indicate a preference for palliative care closer to home, it may also indicate a need for improved telemedicine infrastructure for patients with palliative needs. Although telemedicine may hold promise for increasing access to care for disadvantaged groups, intentional effort may be needed to best reach these groups.

Understanding treatment yield for IPPs and VPs was a primary and novel objective of this study. Our VPs had significantly lower yield than IPPs. VPs with GU diagnoses had the largest reduction in yield compared with IPPs, likely attributed to the decreased acuity and favorable natural history of most prostate cancers that allow for the opportunity to seek multiple medical opinions.^23^ VPs who underwent treatment lived closer than those who did not; treated VPs still lived significantly farther away than treated IPPs. This shows telemedicine's ability to reach patients who may have faced challenges accessing care or those seeking a second opinion.

With lower ADI in VPs, it could be suggested that intrapandemic telemedicine reached those considered to be less health care–deprived. However, this may be explained by the unexpected increase in demand for telemedicine, which limited direct efforts to reach the medically underprivileged. With increased ADIs among VPs undergoing treatment, we show potential to expand oncology care access to patients who may not have radiotherapy near their home, including those who live in rural and/or health care–deprived areas. Most of our catchment area is considered rural and the majority of those living more than 100 miles away have high ADIs. As telemedicine becomes a more integral part of daily practice, improved identification of underserved populations, those needing telemedicine infrastructure, and those who would benefit from specialty radiotherapy is warranted.

A unique aspect of our institution compared with other regional radiation oncology centers is the availability of specialty radiotherapy services such as proton radiotherapy, brachytherapy, and access to a large comprehensive cancer center with clinical trial availability. VPs who underwent treatment used proton radiotherapy 1.9 times more than IPPs. This difference could be explained by significant increases in proton therapy utilization for those with CNS, head/neck/skin, sarcoma, GU, and lymphoma diagnoses, all sites in which proton therapy indications are merited and/or rising. This suggests that the availability of a unique treatment modality may have prompted a greater conversion from consultation to treatment or that consultation was performed for review of indications for proton therapy. This highlights the potential of telemedicine, with improved infrastructure, to improve access to specialty radiation oncology care.

This study has several limitations. Our center is a large tertiary cancer center with subspecialized care and treatment modalities, which may not reflect the general radiation oncology practice. Also, our patient population is generally homogenous in race. Therefore, it may not discern telemedicine and care access challenges faced by racial or ethnic minorities. Although our expanded virtual reach leaned in favor of insured, more privileged patients, we were able to treat patients further from clinic with a higher ADI and increase the access to specialized radiotherapy techniques using telemedicine services. Expansion of telemedicine to underprivileged populations is warranted, and a broader study of telemedicine in radiation oncology would benefit from increased racial, gender, and geographic diversity. In determining treatment yield, we only assessed patients who underwent radiotherapy at our center. Some patients may not have been recommended radiotherapy and the proportion may have differed between IP and VPs. Also, patients may have undergone consultation within the study window but initiated radiotherapy outside of the December 2021 study cutoff date. As a result of these factors, the actual treatment yield may have been higher than reported.

Our study did not investigate the patient attitudes toward telemedicine, their ability to use telemedicine interfaces, and how attitudes may have changed due to the pandemic. Although studies show general patient satisfaction and equivalent care outcomes with telemedicine,^24-27^ assessment of patient satisfaction with virtual technologies is merited for continued improvement in quality. Similarly, the effect of telemedicine on cancer care and outcomes is worthy of continued investigation. Previous studies have shown many socioeconomic metrics to be linked to decreased access to care during the COVID-19 pandemic; exploration of other indices would allow for better understanding of patient and technological infrastructure needs.^14-17^ Development of tools to reach patients with limited access and/or specialty care needs is warranted.

Telemedicine in radiation oncology is a developing resource with potential to enhance cancer care. Since the novelty and alarm of the COVID-19 pandemic have subsided, telemedicine has transformed from a necessity to a powerful tool that can enhance access to medical care. Telemedicine can help increase the reach of subspecialty care within radiation oncology, a technical and procedural field, and as a result increase access to other technical medical specialties. Further research is needed to improve telemedicine outreach and infrastructure.

## APPENDIX

**TABLE A1.** ADI for IP Visits by Yield

ADI Characteristic	Treatment (N = 8,211)		
	Yes (n = 5,883)	No (n = 2,328)	*P*
ADI, No. (%)			
Available	5,414 (92.0)	2,142 (0.0)	
US, unavailable	371 (6.3)	147 (1.8)	
International	98 (1.7)	39 (0.5)	
ADI, national percentage			
Mean (SD)	49.8 (22.3)	48.5 (22.5)	.02

Abbreviations: ADI, area deprivation index; IP, in-person; SD, standard deviation.

**TABLE A2.** ADI for Virtual Visits by Yield

ADI Characteristic	Treatment (N = 2,143)		
	Yes (n = 1,026)	No (n = 1,117)	*P*
ADI, No. (%)			
Available	938 (91.4)	962 (86.1)	
US, unavailable	83 (8.1)	125 (11.2)	
International	5 (0.5)	30 (2.7)	
ADI, national percentage			
Mean (SD)	46.1 (22.9)	42.9 (24.9)	.004

Abbreviations: ADI, area deprivation index; SD, standard deviation.
